# The effect of nurse empowerment educational program on patient safety culture: a randomized controlled trial

**DOI:** 10.1186/s12909-018-1255-6

**Published:** 2018-07-03

**Authors:** Maryam Amiri, Zahra Khademian, Reza Nikandish

**Affiliations:** 1 Department of Nursing, School of Nursing and Midwifery, Shiraz University of Medical Sciences, Shiraz, Iran; 2 Anesthesia and Critical Care Emergency Medicine Department, Namazi Hospital, Shiraz University of Medical Sciences, Shiraz, Iran

**Keywords:** Culture, Intensive care units, Nursing, Supervisory, Nurses, Patient safety, Patient safety culture, Safety

## Abstract

**Background:**

The complexity of patients’ condition and treatment processes in intensive care units (ICUs) predisposes patients to more hazardous events. Effective patient safety culture is related to lowering the rate of patients’ complications and fewer adverse events. The present study aimed to determine the effect of empowering nurses and supervisors through an educational program on patient safety culture in adult ICUs.

**Methods:**

A randomized controlled trial was conducted during April–September 2015 in 6 adult ICUs at Namazi Hospital, Shiraz, Iran. A total of 60 nurses and 20 supervisors were selected through proportional stratified sampling and census, respectively, and randomly assigned to the experimental and control groups. The intervention consisted of a two-day workshop, hanging posters, and distributing pamphlets that covered topics such as patient safety, patient safety culture, speak up about safety issues, and the skills of Team Strategies and Tools to Enhance Performance and Patient Safety. Data were collected through a hospital survey on patient safety culture. Eventually, 61 participants completed the study. Data were analyzed using descriptive statistics, independent-samples t-test, paired-samples t-test, and Chi-square test. *P* < 0.05 was considered statistically significant.

**Results:**

In the experimental group, the total post-test mean scores of the patient safety culture (3.46 ± 0.26) was significantly higher than that of the control group (2.84 ± 0.37, *P* < 0.001). It was also higher than that of the pre-test (2.91 ± 0.4, *P* < 0.001). Additionally, significant improvements were observed in 5 out of 12 dimensions in the experimental group. However, dimensions such as non-punitive response to errors and the events reported did not improve significantly.

**Conclusion:**

Empowering nurses and supervisors could improve the overall patient safety culture. Nonetheless, additional actions are required to improve areas such as reporting the events and non-punitive response to errors.

**Trial registration:**

IRCT2015053122494N1. Date registered: March 2, 2016.

## Background

Patient safety is an important element in offering high-quality health care services. However, it is estimated that approximately 400,000 annual deaths are related to preventable harms [1]. The complexity of patients’ condition and treatment processes in Intensive Care Unit (ICU) predisposes patients to more hazardous events [2]. In a prospective study, during 2013–2014, the rate of adverse events per 1000 patient-days in an ICU was 80.5 in which 45% were preventable [3]. The epidemiology of medical errors in Iran is ambiguous. Zargarzadeh has estimated that 24,500 annual deaths are related to medical errors [4]. In addition, in an ICU, among 307 medication doses, 214 (69.7%) errors were identified during administration (*n* = 132, 42.99%), prescription (*n* = 74, 24.1%), and transcription (*n* = 8, 2.61%) of medications [5]. Moreover, 48 medication errors per 100 orders were observed in a pediatric ICU [6].

Poor communication and collaboration [7], lack of knowledge, and inadequate training were among the main causes of nursing errors in ICUs [8]. Studies have shown the lack of communication skills in nurses and nursing students [9, 10]. Hence, a training program for nurses on patient safety alongside with strategies to improve professional communication is required to improve patient safety.

High mortality and morbidity associated with medical errors indicate the importance of promoting patient safety in critical care units. Nurses play a key role in improving patient safety due to their continuous presence at patients’ bedsides and interaction with their families and other healthcare professionals [11]. For instance, critical care unit nurses have often reported that they identified and corrected errors such as medication and procedural errors related to nurses and other caregivers [12]. Henneman et al. identified multiple strategies to identify the patient, recognize other team members, and the plan of care, which nurses used to detect, discontinue, and correct errors in critical care settings [13].

Research findings indicated that a strong patient safety culture is associated with a lower rate of patients’ complications and fewer adverse events [14, 15]. It is defined as a culture whereby nurses are aware of errors and are encouraged to discuss them. This, in turn, improves their ability to learn from past mistakes and take corrective measures [16].

A meta-analysis, including 11 descriptive studies on hospital staff, showed that only 8.3 and 32.3% of the respondents of the reviewed articles have rated patient safety culture in Iran as excellent and very good, respectively [17]. The important role of patient safety culture necessitates improvement of these strategies in clinical settings. Nevertheless, interventions that may improve patient safety culture are not adequately defined [18]. In a study, the positive effects of some interventions, such as executive walk rounds [19] and the role of nurse managers in regular assessment and support of the safety culture were reported [20]. Consequently, the participation of nurse managers in the planning and implementation of strategies, to improve patient safety culture, may reinforce these strategies [18].

Several studies have reported the effects of nurse empowerment interventions on patient safety culture. A type of strategy is an educational program, such as online module, addressing patient safety which increases positive scores of nurses in two dimensions of patient safety culture (i.e. “non-punitive response to errors” and “frequency of event reporting”) [21]. Teaching teamwork also improves staff perception of patient safety culture in the emergency department [22]. Another empowerment strategy is to encourage nurses to speak up. Sayre (2010) reported that nurses behavior towards patient safety protection increased when encouraged to speak up in a situation of a threat to patient safety [23].

In order to improve the quality of care and patient safety, the Institute of Medicine (2003) recommended a reform in health profession education [24]. Accordingly, the Quality and Safety Education for Nurses (QSEN) project was introduced to train nurses on the required competencies to improve the quality of care and patient safety [25]. Considering the important role of nurses and leaders in ensuring patient safety and in providing a strong patient safety culture, we developed and studied the effects of an innovative empowerment program on patient safety culture. This program is unique in a sense that it involves nurses and supervisors with an integrated exclusive educational program which encourages them to speak up. The present study aimed to determine the effect of empowering nurses and supervisors through an educational program on patient safety culture in adult ICUs.

## Methods

**Fig. 1:**
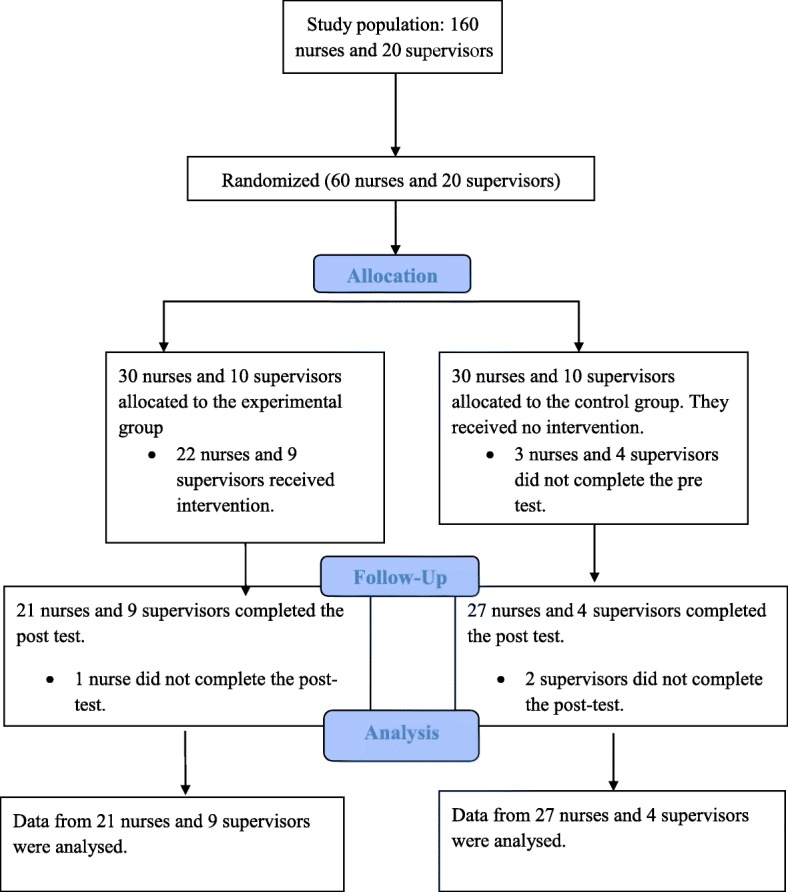
The CONSORT diagram

This randomized controlled trial with a pre-test and post-test control groups was conducted during April–September 2015 in 6 adult ICUs at Namazi Hospital, Shiraz, Iran. All the above-mentioned ICUs were similar in terms of patient safety policies. The study population included 160 nurses and 20 supervisors. The nurse:patient ratio in these wards was 1:2. The sample size consisted of 60 nurses and 20 supervisors. The nurses were selected based on proportional stratified sampling. Therefore, the number of selected nurses from each ICU was proportional to the total number of its nurses. Supervisors were nurses with at least a Bachelor’s degree and responsible for oversight nursing services in the studied ICUs. Note that the supervisors did not provide direct patient care. All supervisors at the hospital participated in the study. To randomly allocate nurses, a number was assigned to each ICU and categorized into the control and experimental groups, based on permuted block randomization. In total, 30 nurses from ICUs number 1, 3, and 6 (surgical, neurosurgical, and general ICU) were assigned to the experimental group. In addition, 30 nurses from ICUs number 2, 4, and 5 (medical, neurosurgical, and general ICU) were assigned to the control group. Based on permuted block randomization, all supervisors at the hospital were assigned to the experimental (*n* = 10) and control (*n* = 10) groups. The experimental group, including 30 nurses (ICUs number 1, 3, and 6) and 10 supervisors received the educational empowerment program. The control group included 30 nurses (ICUs number 2, 4, and 5) and 10 supervisors that did not receive any intervention. The inclusion criteria were having at least 6 months experience in an adult ICU and at least a Bachelor’s degree in nursing. The exclusion criteria were the unwillingness to participate, failure to complete the pre-test, and lack of participation in training sessions. A total of 61 out of 80 individuals (experimental group: *n* = 30, control group: *n* = 31) completed the post-test questionnaire (Fig. 1).

### The educational empowerment program

The educational empowerment program was carried out by one of the researchers. This program started with a two-day workshop (8 h), followed by hanging posters and handing out educational pamphlets to the nurses and supervisors of the experimental group at their workplace. The educational contents of the workshop, posters, and pamphlets were matched. The workshop included education on patient safety, patient safety culture, speak out in a situation of a threat to patient safety, and the skills of Team Strategies and Tools to Enhance Performance and Patient Safety (TeamSTEPPS). TeamSTEPPS was developed by the Agency for Healthcare Research and Quality (AHRQ) to improve patient outcomes. It included communication, leadership, mutual support, and situational monitoring skills [26]. The workshop consisted of a lecture, group discussion, and presenting scenarios. In addition, some textual and graphical posters (related to TeamSTEPPS skills, speak up, and patient safety culture) were placed on the walls of patient’s unit in the ICUs of the experimental group for a period of 6 weeks. During the following 6 weeks, every week one pamphlet was handed out to the nurses in the experimental groups. Pamphlets contents included communication, mutual support, situation monitoring, leadership, speak up, and patient safety culture.

### Data collection

Data were collected using the Persian version of Hospital Survey on Patient Safety Culture (HSOPSC) developed by the AHRQ. The validity of the HSOPSC in Iran was verified by 15 experts and its reliability measured by Cronbach’s alpha coefficient (0.84) [27]. This questionnaire has 42 items in 12 dimensions. These dimensions include: teamwork within units, manager expectations and actions promoting patient safety, organizational learning and continuous improvement, management support for patient safety; overall perception of patient safety, feedback and communication on errors, communication openness, frequency of events reported; teamwork across hospital units, staffing, handoffs and transitions, non-punitive response to errors. The items were answered on a five-point Likert scale, from completely disagree (1) to completely agree (5) or from never (1) to always (5). There were a few negatively worded items in the questionnaire that were reverse coded. If the proportion of respondents who answered “completely agree”/“agree”, or “always”/“most of the time” on each item was more than 50%, this was considered as strong, otherwise (below 50%) as the weak point of the safety culture. In addition to these 42 questions, there was a single item on patient safety grading in the unit. This item was answered on a five-point Likert scale from excellent (score = 5) to failing (score = 1) and was analyzed separately as a single item [20, 28]. The pre-test was completed individually before the workshop. Three months after the workshop, the post-test was conducted individually in both groups.

### Data analysis

Statistical analysis was carried out using the SPSS software version 18.0. The results of One-Sample Kolmogorov-Smirnov showed normal distribution of data before (*P* = 0.72) and after (*P* = 0.96) the intervention, except for the single item on patient safety grading. Descriptive statistics was used to describe age, sex, education, position, and the total scores of the patient safety culture and its dimensions. To compare the mean scores between the two groups and within each group, the independent-samples t-test and paired-samples t-test were used. The single item on patient safety grading was compared between the control and experimental groups using the Mann-Whitney test. This item was compared before and after the intervention in each group using the Wilcoxon test. The effect size for paired t-test was calculated by the Cohen (1988) equation as follows:

$$ \mathrm{Effect}\ \mathrm{size}=\mathrm{d}=\frac{\mathrm{M}1\hbox{-} \mathrm{M}2}{\mathrm{S}\ \mathrm{pooled}},\kern1.25em {\mathrm{S}}_{\mathrm{pooled}}=\sqrt{\frac{\mathrm{S}{1}^2+\mathrm{S}{2}^2}{2}} $$

Where M1 and M2 are post-test means of the experimental and control groups, respectively. Spooled: Pooled standard deviation, and S1 and S2: Post-test standard deviations of the experimental and control groups, respectively.

The effect size of 0.2, 0.5, and 0.8 was considered small, medium, and large, respectively [29]. *P* < 0.05 was considered statistically significant.

## Results

The sample size included 48 nurses and 13 supervisors. The experimental and control groups were homogeneous in terms of age, sex, marital status, education, and position (Table 1).

**Table 1:** Distribution of demographic characteristics of the participants

Group		Experimental	Control	Total	*P*-value
Demographic characteristics		Mean (±SD)	Mean (±SD)	Mean(±SD)	
Age		34.87 (±7.8)	36.06 (±8.03)	33.46(±7.91)	0.79
		Frequency (%)	Frequency (%)	Frequency (%)	
Sex	Female	27 (90)	26 (83.8)	53 (86.9)	0.48
Education	Bachelor’s degree	26 (86.7)	30 (96.8)	56 (91.8)	0.15
	Master’s degree	4 (13.3%)	1 (3.2%)	5 (8.2)	
Position	Nurse	21 (70)	27 (87.1)	48 (78.68)	0.27
	Supervisor	9 (30)	4 (12.9)	13 (21.32)	

Table 2: The response of all participants, both in the experimental and control groups, on patient safety culture prior to the intervention. The findings showed that before the intervention, the organizational learning and continuous improvement (72.46% of positive responses) and staffing (9.95% of positive responses) were the strongest and the weakest dimensions of patient safety culture (Table 2).

**Table 2:** The mean, standard deviation, and percentage of positive responses to the 12 dimensions of patient safety culture by all participants before the intervention

Dimensions	Mean (±SD)	Percent (%)
Teamwork within units	2.71 (±0.8)	45.65
Manager expectations and actions promoting patient safety	3.39 (±0.75)	59.9
Organizational learning and continuous improvement	3.65 (±0.73)	72.46
Management support for patient safety	3.08 (±1.04)	55.53
Feedback and communication on errors	3.39 (±0.81)	60.86
Communication openness	2.77 (±0.72)	23.27
Frequency of events reported	2.77 (±0.62)	26.46
Teamwork across hospital units	3.08 (±0.84)	53.2
Staffing	1.76 (±0.54)	9.95
Handoffs and transitions	2.56 (±0.86)	28.15
Non-punitive response to errors	2.36 (±1.03)	21.66
Overall perception of patient safety	3.08 (±0.66)	51.2
Total scores of the patient safety culture	2.88 (±0.38)	42.35

The pre-test means of the experimental and control groups of the total scores of patient safety culture and its dimensions were not statistically different. However, in the experimental group, the total post-test mean scores of patient safety culture was significantly higher than that of the control group (3.46 ± 0.26 vs. 2.84 ± 0.37, *P* < 0.001), and it was also higher than that of the pre-test (3.46 ± 0.26 vs. 2.91 ± 0.4, *P* < 0.001, effect size (*d)* = 1.94). In addition, significant improvements were observed in 5 out of 12 dimensions in the experimental group. The mean scores of teamwork within units (3.95 ± 0.43 vs. 2.91 ± 0.74, *P* < 0.001, *d* = 1.03), manager expectations and actions promoting patient safety (4.22 ± 0.31 vs. 3.48 ± 0.83, *P* < 0.001, *d* = 0.84)**,** and organizational learning and continuous improvement (4.45 ± 0.45 vs. 3.83 ± 0.65, *P* < 0.001, *d* = 0.83) increased significantly in the experimental group. Furthermore, the post-test means of communication openness (4.22 ± 0.44 vs. 2.72 ± 0.67, *P* < 0.001, *d* = 1.82) and handoffs and transitions (4.23 ± 0.69 vs. 2.75 ± 0.9, P < 0.001, *d* = 1.30) increased significantly in the experimental group. However, there was no significant change in the control group mean scores (Table 3).

**Table 3:** Comparison of patient safety culture before and after the intervention within and between groups

Dimensions	Group^a^	Pre-test Mean (±SD)	Post-test Mean (±SD)	P-value (within group)
Teamwork within units	Experimental	2.91(**±**0.74)	3.95(**±**0.43)	**< 0.001**
	Control	2.51 (**±** 0.82)	2.69(**±**0.80)	0.4
	P-value	0.06	**< 0.001**	
Manager expectations and actions promoting patient safety	Experimental	3.48 (**±**0.83)	4.22 (±0.31)	**< 0.001**
	Control	3.22 (±0.68)	3.23 (±0.76)	0.5
	P-value	0.3	**< 0.001**	
Organizational learning and continuous improvement	Experimental	3.83 (±0.65)	4.45 (±0.45)	**< 0.001**
	Control	3.49 (±0.82)	3.13 (±0.86)	0.1
	P-value	0.06	**< 0.001**	
Management support for patient safety	Experimental	3.15 (±1.05)	3.26 (±0.94)	0.5
	Control	2.97 (±1.04)	3.31 (±0.99)	0.1
	P-value	0.6	0.8	
Overall perception of patient safety	Experimental	2.92 (±0.62)	3.08 (±0.53)	0.1
	Control	3.29 (±0.63)	3.23 (±0.73)	0.6
	P-value	0.06	0.3	
Feedback and communication on errors	Experimental	3.25 (±0.85)	3.56 (±0.72)	0.1
	Control	3.53 (±0.78)	3.52 (±0.77)	0.9
	P-value	0.2	0.8	
Communication openness	Experimental	2.72 (±0.67)	4.22 (±0.44)	**< 0.001**
	Control	2.80 (±0.79)	2.51 (±0.74)	0.1
	P-value	0.5	**< 0.001**	
Frequency of events reported	Experimental	2.91 (±0.56)	2.76 (±1.04)	0.4
	Control	2.66 (±0.66)	2.51 (±0.68)	0.2
	P-value	0.09	0.2	
Teamwork across hospital units	Experimental	2.94 (±0.93)	3.06 (±0.84)	0.5
	Control	3.17 (±0.76)	3.15 (±0.81)	0.8
	P-value	0.1	0.6	
Staffing	Experimental	1.84 (±0.62)	1.97 (±0.52)	0.3
	Control	1.69 (±0.46)	1.68 (±0.57)	0.9
	P-value	0.2	**0.04**	
Handoffs and transitions	Experimental	2.75 (±0.91)	4.23 (±0.69)	**< 0.001**
	Control	2.42 (±0.80)	2.69 (±0.66)	0.2
	P-value	0.1	**< 0.001**	
Non-punitive response to errors	Experimental	2.25 (±0.93)	2.78 (±0.94)	**0.02**
	Control	2.45 (±1.15)	2.46 (±1.17)	0.9
	P-value	0.4	0.2	
Total scores of the patient safety culture	Experimental	2.91 (±0.4)	3.46 (±0.26)	**< 0.001** ^**b**^
	Control	2.86 (±0.37)	2.84 (±0.37)	0.8
	P-value	0.5	**< 0.001**	
Safety score	Experimental	2.63 (±0.7)	3.37 (±0.5)	**< 0.001**
	Control	2.88 (±0.4)	2.90 (±0.5)	1.0
	P-value	0.07	**0.002**	

^a^The between groups *P*-value is provided

^b^The effect size of total scores of the patient safety culture is 1.94

The bold numbers are significant *p*-values

## Discussion

In the present study, the effect of an innovative educational empowerment program on patient safety culture is investigated. The finding suggests that the empowerment program improved the total score of patient safety culture. The effect size of this score was large (1.94) [29]. This shows that the effect of the intervention is strong and clinically important.

The results of the present study showed that communication openness improved after the intervention. This domain indicates member’s ability to question decisions and actions of individuals with more authority and the ability to speak up when there is a concern about patient safety. This finding was in line with the findings of a study by Andreoli et al. in which SBAR was used to communicate and manage fall risk, [30] and also by Khademian et al. in which the anesthesia and operating room nursing students’ perceptions of communication dimension improved after TeamSTEPPS training [31]. However, it was in contradiction with the results of two other studies in which patient safety education and teamwork training of nurses and hospital staff did not improve their attitudes on communication openness [21, 22]. In the current study, one aspect of the intervention was training in speaking up, which may explain the differences between the current findings and those from previous studies. Evidence show that hospital staff are not competent enough in speaking up. This is based on the fact that, among the 447,584 hospital staff in the United States, 65% of the respondents stated that they were afraid of asking questions when they felt something was wrong [20]. Iranian nurses noted a sense of powerlessness, due to dominance by the medical staff, prevents them from talking in favor of their patients [32]. Therefore, empowering nurses to speak up might help them to overcome these barriers.

In the pre-test, dimensions of teamwork within units and handoffs and transition were the weak aspects of patient safety culture. However, after the intervention, some improvements were observed in the experimental group and these were elevated to the strong dimensions. Similar results in previous studies have shown that training teamwork skills, using SBAR tool, and interventions based on HSOPSC domains enhanced teamwork within units [30, 33, 34]. However, in some other studies, no improvement was achieved after training [21, 22]. Similar to our findings, other studies showed improvement on handoffs and transitions [22, 30]. Therefore, we could suggest a similar empowerment program to improve teamwork within units and handoff and transitions.

In the present study, “teamwork across the units” did not improve significantly after the intervention. We involved supervisors in addition to nurses in the empowerment program to reinforce their role in patient safety culture improvement. We expected that empowering supervisors would improve coordination and teamwork across units. These findings may be related to the small sample size of supervisors. We should bear in the mind that this dimension was strong before the intervention; however, we expected more improvement. Similarly, in other studies in which education was the main intervention, “teamwork across the units” did not improve significantly [21, 22].

The dimensions of “non-punitive response to errors” and “the frequency of events reported” were among the weakest dimensions of patient safety culture before the intervention. The mean scores of “non-punitive response to errors” after the intervention had significantly increased in the experimental group. However, these scores were not significantly different to that of the control group. Therefore, we could not conclude that this dimension improved due to the intervention. In addition, the frequency of events reported did not show any improvement. In a previous study, “non-punitive response to errors” had improved while “the frequency of events reported” did not improve [30]. In another study, using a single group pre-test post-test design, the only two dimensions that had improved after safety training were “non-punitive response to errors” and “the frequency of events reported” [21]. Consequently, based on the current results, we could not conclude that education can improve non-punitive response to errors. Therefore, there is a need for collaboration among all team members and leaders towards problem-solving and to increase the number of events being reported.

It seems that the involvement of nurses and supervisors in the empowerment program was not sufficient to improve three important dimensions: staffing, error reporting, and non-punitive response to errors. Therefore, we recommend that in the future higher-level hospital executives should also be involved in empowerment programs.

### Limitations of the study

The main limitation of the present study was related to the use of a self-reported instrument in order to explore the effects of empowerment on the patient safety culture. It is recommended that further studies should be conducted using observational data collection methods. Additionally, studies that assess the viewpoints of other parties such as patients are recommended.

## Conclusion

This innovative empowerment program which involved nurses and supervisors resulted in improved patient safety culture scores and development in some dimensions. Communication openness, handoffs and transitions, teamwork within units, learning and continuous improvement, manager’s expectations and actions promoting patient safety improved significantly after the intervention. Therefore, this program can be utilized to promote these important dimensions of patient safety culture. However, dimensions such as staffing, “non-punitive response to errors”, and “frequency of events that were reported” continued to be the weak domains of the patient safety culture throughout the study. Thus, to improve these dimensions, conducting long-term studies and additional actions are also required. Given the importance of reporting errors and adequate staffing in improving patient safety, it is recommended that these items should be considered as a top priority for healthcare managers and hospital policymakers.

## Abbreviations

AHRQ: Agency for Healthcare Research and Quality
HSOPSC: Hospital Survey on Patient Safety Culture
ICU: Intensive care units
SPSS: Statistical Package for Social Science
TeamSTEPPS: Team Strategies and Tools to Enhance Performance and Patient Safety
